# Management of children with auditory neuropathy spectrum disorder (ANSD)^☆^

**DOI:** 10.1016/j.bjorl.2015.08.022

**Published:** 2015-12-18

**Authors:** Çağıl Gökdoğan, Şenay Altınyay, Bülent Gündüz, Yusuf Kemal Kemaloğlu, Yıldırım Bayazıt, Kemal Uygur

**Affiliations:** a Gazi University Hospital, Department of Audiology, Ankara, Turkey; b Gazi University Hospital, Department of ENT-HNS, Ankara, Turkey

**Keywords:** Auditory neuropathy spectrum disorders, Sensorineural hearing loss, Amplification, Rehabilitaton, Espectro da neuropatia auditiva, Perda auditiva neurossensorial, Amplificação, Reabilitação

## Abstract

**Introduction:**

ANSD is a challenging problem.

**Objective:**

To present our experience on management of the children with ANSD with respect to clinical data.

**Methods:**

This retrospective study included all children younger than 16 years of age who applied to the department between 2005 and 2013 (with the exception of newborn hearing screening NHS referrals). The data were derived from pure tone, OAEs and ABR tests, and further medical risk factors of the subjects were evaluated.

**Results:**

ANSD was recognized in 74 ears of 40 children (B/U: 34/6) among 1952 children with SNHL (2.04%) detected among 9520 applicants to the department (0.42%). The clinical tests revealed that hearing loss greater than 15 dB was present in both ears of 38 cases. The degree of hearing loss was profound in 48% children, severe in 12% children, moderate in 28% children, mild in 10% children and normal in 5% children. ABRs were absent/abnormal in 37/3 ears and CMs were detected in all. Acoustic reflexes were absent in all ears. Rehabilitation was managed by CI and hearing aids in 15 and 23 cases, respectively. FM system was given to two cases displaying normal hearing but poor speech discrimination in noisy environments.

**Conclusion:**

ANSD is a relatively challenging problem for the audiology departments because of its various clinical features and difficulties in management. Our patients with ANSD most commonly displayed profound hearing loss. The number of overlooked cases may be minimized by performing ABR and OAE in every case referred with the suspicion of hearing loss.

## Resumo

**Introdução:**

Espectro da neuropatia auditiva ainda é uma condição clínica desafiadora.

**Objetivo:**

Apresentar nossa experiência no tratamento de crianças com espectro da neuropatia auditiva em relação aos dados clínicos.

**Método:**

Este estudo retrospectivo incluiu crianças menores de 16 anos de idade que deram entrada no departamento entre 2005 e 2013 (com exceção de encaminhamentos para triagem auditiva neonatal). Foram avaliados os dados obtidos a partir dos exames de audiometria tonal, emissões otoacústicas (EOA), potencial evocado auditivo de tronco encefálico (ABR) e outros fatores de risco.

**Resultados:**

Das 1.952 crianças com perda auditiva neurossensorial (2,04%) detectadas dentre os 9.520 candidatos que deram entrada no departamento (0,42%), espectro da neuropatia auditiva foi reconhecida em 74 orelhas de 40 crianças (B/U: 34/6). Os testes clínicos revelaram que uma perda auditiva superior a 15 dB estava presente em ambas as orelhas em 38 casos. O grau de perda auditiva das crianças era profundo em 48%, grave em 12%, moderado em 28%, leve em 10%, e normal em 5%. ABR estava ausente/anormal em 37/3 orelhas e microfonia coclear foi detectado em todas as crianças. Reflexos acústicos estavam ausentes em todas as orelhas. A reabilitação foi tratada com implante coclear e aparelhos auditivos em 15 e 23 casos, respectivamente. Um sistema FM foi utilizado em dois casos que apresentavam audição normal, mas discriminação deficiente da fala em ambientes ruidosos.

**Conclusão:**

Espectro da neuropatia auditiva é um problema desafiador para os departamentos de audiologia, devido às suas várias características clínicas e dificuldades no tratamento. Em nossos pacientes a perda auditiva profunda foi a mais frequente. O número de casos negligenciados pode ser diminuído com a realização dos exames ABR e EOA em todos os casos encaminhados com suspeita de perda auditiva.

## Introduction

The hearing loss known as auditory neuropathy spectrum disorder (ANSD) has been described by the presence of otoacoustic emissions despite absent or severely abnormal auditory brainstem responses (ABRs).^1^, ^2^, ^3^ Foerst et al. reported its prevalence as 0.94% and 8.44% for infants at risk for hearing impairment and profoundly hearing impaired children, respectively.^4^ Previously, Kraus et al. determined these rates as 1.3% and 14%.^5^

It has been reported that ANSD was related with various clinical and audiological patterns. Pure tone thresholds (PTAs) range from normal or near normal to severe hearing loss, particularly characterized by impaired auditory processing skills in noisy environments. These subjects present very low speech discrimination scores (SDS) which are not associated with the pure tone levels. Acoustic reflexes are absent in the majority of the cases.^1^, ^2^, ^6^

It has been reported that, particularly because of poor SDS in relation to better PTAs, management process of children with ANSD is more problematic than that of children with other hearing loss patterns.^7^, ^8^, ^9^, ^10^ Furthermore, since the site of the lesion in the subjects clinically collected into the ANSD group is still unknown and there has not been a test to discriminate the lesion site of the given cases,^2^ selection of the management option becomes more difficult.^7^, ^8^, ^9^, ^10^ Major interests at this point have been focused on whether the cochlear implant (CI) is beneficial or not in the given cases. However, in some cases, it could even be difficult to decide between the options of a hearing device and “waiting and actively observing”.^7^, ^8^, ^9^, ^10^ The accepted approach toward children with ANSD is to initially provide amplification using hearing aids; however, many ANSD patients demonstrate little functional hearing and speech understanding with conventional amplification. In subjects who demonstrate poor speech understanding and delayed language development with hearing aids, cochlear implantation (CI) may be offered.^2^, ^11^

Our purpose in this study is to present our experience on the management of children with ANSD with respect to clinical data.

## Methods

This study has been done in a retrospective manner and included all children younger than 16 years of age, who applied to the audiology department between 2005 and 2013 (Ethical Committee: 446). The subjects were first divided into two subgroups according to presence of sensorineural hearing loss (SNHL) (>15 dB),^11^ and then the SNHL group was divided into those with ANSD and the others. Thus, prevalence of ANSD for the children applying to the audiology department and prevalence of ANSD in those with SNHL were calculated.

The audiological evaluation of patients who are suspected of having ANSD should include the following criteria: presence of CM; abnormal or absent ABR.

The audiometric data of the subjects with ANSD and auditory perception skills – language development were evaluated.

### Audiometric tests

Age-specific pure tone audiometry from 250 to 6000 Hz was performed for all subjects using conversional, play or behavioral methods. Speech detection and recognition assessments included speech awareness test (SAT) for children and speech recognition threshold. Tympanometry and acoustic reflex evaluation were also performed at the time of testing. Acoustic reflex thresholds were measured ipsilateral and contralateral with pure tone stimuli from 500 to 4000 Hz. Stimulated ears were considered absent when there was no response to test intensities up to 110 dB HL.

### Otoacoustic emissions (OAE)

Click evoked otoacoustic emissions were measured with an ILO – 92 OAE system. The click level ranged from 80 to 86 dB peak sound pressure. Responses to as many as 260 stimuli were averaged over a 20 ms window and stored in two separate buffers. The presence of normal transient evoked otoacoustic emissions was determined by response amplitude of at least 3 dB and waveform reproducibility in at least three octave bands of >75%.

### Auditory brainstem response (ABR)

ABR was recorded in a single electrode configuration, in a channel running from the forehead to the ipsilateral ear using a band-pass filter between 100 and 3000 Hz. Click stimuli consisted of one run of condensation followed by one run rarefaction clicks presented monaurally at rates of 13.0 per second and at intensities of 75 dB and, when necessary, at 95 dB HL. CM was demonstrated by the changing polarity in ABR. Patients underwent assessment in a sound treated room in a state of natural or chloral hydrate induced sleep.

### Auditory perception skills

Auditory perception skills’ evaluation of children with hearing aids and cochlear implants was performed using the Ling sounds test, the Infant-Toddler Meaningful Auditory Integration Scale (IT-MAIS), Meaningful Use of Speech Scale (MUSS) and LittlEARS.

Ling sounds test is an auditory perception skills test and is used to evaluate both the detection and the discrimination of sounds. Six picture cards symbolizing the sounds were used to evaluate children during the test.

The Infant-Toddler Meaningful Auditory Integration Scale (IT-MAIS) is a modification of the Meaningful Auditory Integration Scale (MAIS). It is a structured interview schedule designed to assess the child's spontaneous responses to sound in his/her everyday environment. The assessment is based upon information provided by the child's parent(s) in response to 10 probes. These 10 probes assess three main areas: (1) vocalization behavior, (2) alertness to sounds, and (3) deriving meaning from sound. Specific scoring criteria have been developed for each of the 10 probes.

Meaningful Use of Speech Scale (MUSS) is a parent report scale, which is designed to assess the child's use of speech in everyday situations. It consists of ten inquiries which assess the following areas: vocal control, use of speech without gesture or sign and use of communication strategies in everyday situations.

LittlEARS auditory questionnaire is a parent questionnaire designed to assess the auditory behavior of hearing impaired children who are provided with a CI or a hearing aid prior to their 24th month of life. It records preverbal auditory development during the first two years of hearing in the child's natural environment, taking into consideration reception, understanding and adequate response and vocal-verbal production of acoustic (linguistic) stimuli. The questionnaire consists of 35 age-related sorted questions to be answered with “yes” or “no” by parents.

### Language development evaluation

Direct assessment of expression and receptive language measures included the Preschool Language Scale, version 4 (PLS-4).

### Statistical evaluation

SPSS 16.0 (SPSS, Inc., Chicago, IL, USA) was used. Wilcoxon test was used to compare the auditory perception and language scores of the patients before and after hearing amplification.

## Results

We found that 9520 children were evaluated in the department during the study period and sensorineural hearing loss was recognized in 1912 of them (20.08%). ANSD was found in 74 ears of 40 children (male: 23; female: 17). ANSD prevalence in the children population with sensorineural hearing loss was found to be 1.89%. The average age of children with ANSD at diagnosis was determined as 3.05 ± 2.44 years.

**Figure 1:**
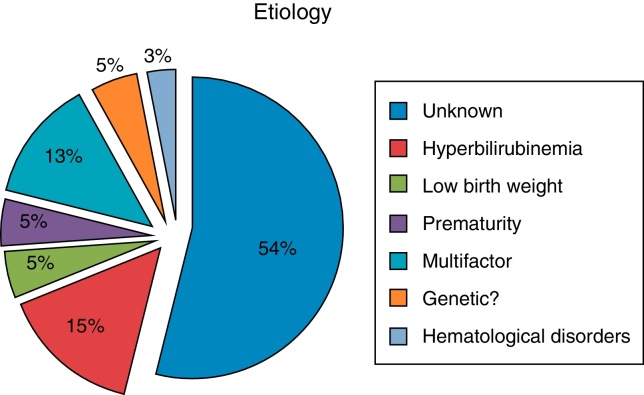
Etiological factors of ANSD patients.

As seen in Fig. 1, factors that are known to be associated ANSD were found in 19 of 40 children; history of hyperbilirubinemia in 15%; prematurity in 5%; low birth weight in 5%; genetic history (siblings) in 5%; 54% of children had no known associated pathology or risk factor.

**Figure 2:**
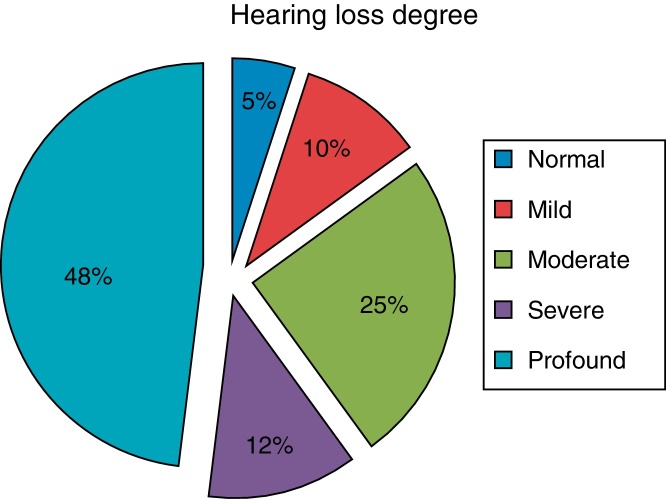
Hearing loss degree of ANSD patients.

All of the ears of the subjects had normal tympanogram and displayed no acoustic reflex response. As seen in Fig. 2, the degree of hearing loss was profound in 48% children, severe in 12% children, moderate in 28% children, mild in 10% children and normal in 5% children.

Transient and distortion OAEs were detected in 32 ears although cochlear microphonics were present in all of the ears with AN. In six subjects with unilateral AN, the contralateral ears had severe-profound hearing loss without cochlear microphonics. In ABR, while only 8% of the patients presented abnormal wave morphology, the remaining ears had no wave-V. Presence of cochlear microphonics was changed by lateralization of ANSD ears.

**Figure 3:**
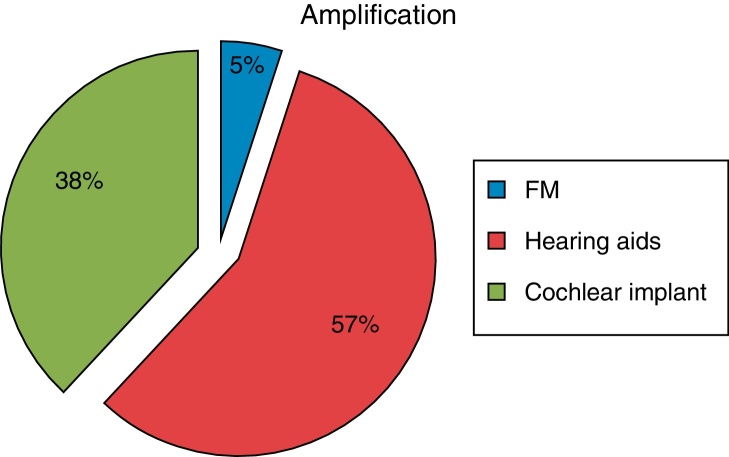
Amplification choices of ANSD patients.

Two patients who had normal hearing were suggested to use the FM system for education. However, both parents of the patients did not wish their children to use them. Thus we did not follow up the FM system's effectiveness in ANSD with normal hearing (Fig. 3).

Twenty-three of the patients were given hearing aids. Two of our patients with hearing aids had no sufficient benefit from its use, but they were not suggested implantation due to their age, irregular hearing aid use and insufficient rehabilitation. Fifteen of the patients with hearing aids did not come to follow-up and rehabilitation.

Fifteen patients with ANSD were suggested cochlear implants and then they were operated on. Intraoperative impedancemetry and neural response telemetry (NRT) were within normal limits. None of them had immediate postoperative complications. Postoperative impedancemetry and neural response telemetry (NRT) were also within normal limits. However, four of the patients with hearing aids did not come to follow-up and rehabilitation.

**Figure 4:**
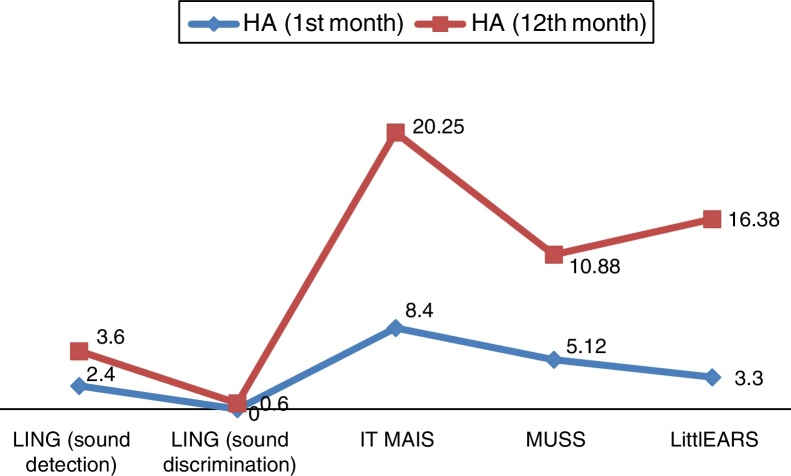
Results of auditory perception skills scores in ANSD with HA.

**Figure 5:**
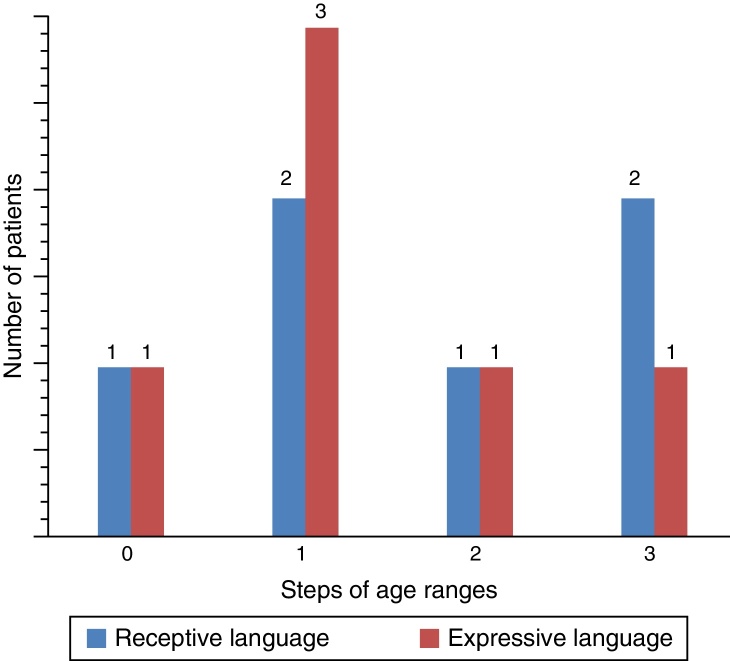
Results of PLS-4 scores in ANSD with HA (PLS-4 scores were shown as progress in age range by steps).

For the evaluation of auditory perception skills LING, IT MAIS, MUSS, and LittlEARS scores were determined one and 12 months after hearing aid fitting, depending on data availability. All patients with sufficient follow-up to date demonstrated significant improvement due to the hearing aids (*p* < 0.05) (Fig. 4). For the evaluation of language development, PLS-4 scores were determined through the fitting of hearing aids one and 12 months after the hearing-aid fitting depending on data availability. All patients with sufficient follow-up to date demonstrated significant improvement due to the hearing aids (*p* < 0.05) (Fig. 5).

**Figure 6:**
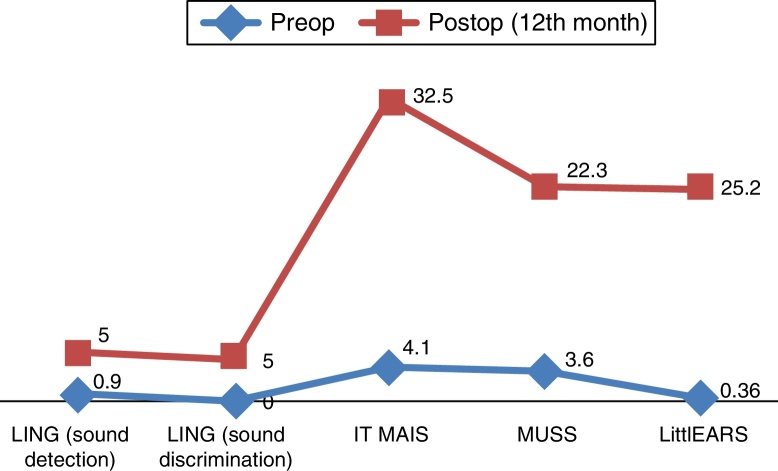
Results of auditory perception skills scores in ANSD with CI.

**Figure 7:**
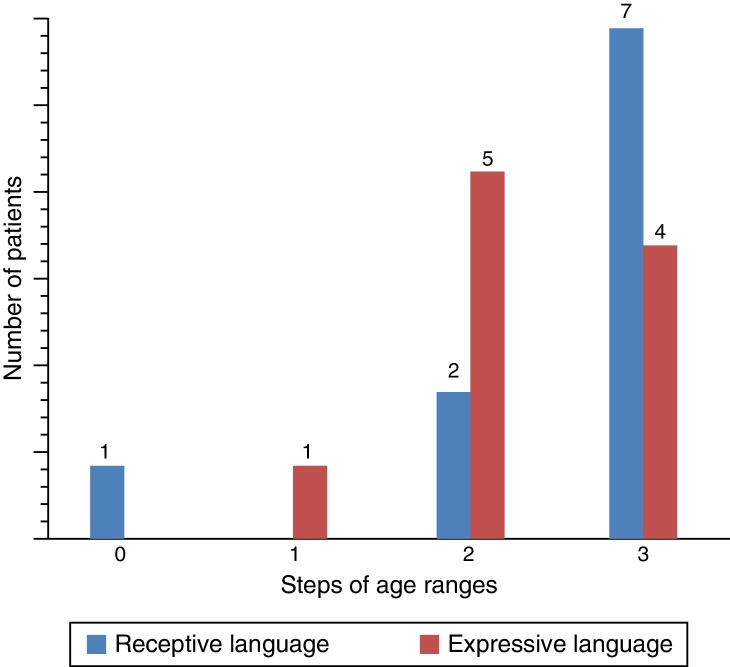
Results of PLS-4 scores in ANSD with CI (PLS-4 scores were shown as progress in age range by steps).

For the evaluation of auditory perception skills LING, IT MAIS, MUSS and LittlEARS scores were determined before the cochlear implantations and 12 months after the cochlear implant fittings depending on data availability. All patients with sufficient follow-up to date demonstrated significant improvement due to the hearing aids (*p* < 0.05) (Fig. 6). For the evaluation of language development, PLS-4 scores were determined before the cochlear implantations and 12 months after the cochlear implantation fitting, depending on data availability. All patients with sufficient follow-up to date demonstrated significant improvement due to the CI (*p* < 0.05) (Fig. 7). However, there was no difference in the effect of ANSD on outcomes between those who use HA and those who use CI (*p* > 0.05).

## Discussion

The term auditory neuropathy was first used by Sininger and colleagues in 1995 and it is currently the most popular term used for the disorder. It should be understood as a consensus term used by researchers to describe the clinical condition of hearing loss characterized by absent ABRs with normal OAEs and CM.^1^

The patients who meet the criteria for the definition of auditory neuropathy represent a heterogeneous population as in our study. In fact, it is quite possible that not all patients with the clinical criteria for this disease have the same pathologic mechanism for their hearing disorder. It is likely that there are various reasons that account for the clinical findings of auditory neuropathy.^3^, ^5^, ^9^, ^12^, ^13^, ^14^, ^15^, ^16^ Also, the patients who were presented in our study had various etiologic or predisposing factors for ANSD. The prevalence was hyperbilirubinemia, detected in 15% of the patients in our study and in 10% to 50% of the reported series.^17^, ^18^, ^19^ Consanguinity is very common in some populations in Turkey. It is associated with a variety of congenital disorders, including hearing loss and ANSD as in our study.^20^ In previous studies, genetic factors have been suspected to be involved in the pathogenesis of AN. Bonfils et al. reported relatives with a dominant inheritance pattern of a progressive hearing loss with characteristics similar to those of ANSD.^21^ Leonardis et al. found a large Gypsy family with hereditary motor and sensory neuropathy associated with ANSD.^22^ Madden et al. hypothesized a recessive inheritance pattern based on the study of three families with two affected children and two other children with family histories positive for hearing loss.^23^ Therefore, in our study, we thought that the two brothers with ANSD could be suffering from a genetic factor concerning their ANSD. Multiple possible etiologies for ANSD have been proposed. An estimated half of all cases are idiopathic.^10^ In our study, the etiology of 54% of the patients with ANSD was not known.

The audiological evaluation of patients who are suspected of having auditory neuropathy must be comprehensive. It should include the following criteria:

1. Hearing loss, usually bilateral, of any degree
2. Presence of OAEs and/or CM
3. Abnormal or absent ABR
4. Poor speech perception
5. Absent acoustic reflexes^23^

The diagnostic hallmark of ANSD is the combination of other normal hair cell activity and abnormal afferent and efferent auditory neural functions probably at the level of the eighth cranial nerve and brainstem. Outer hair cell activation is assessed indirectly by the acoustic energy emitted by the inner ear (OAEs) and the electrical response from the cochlea (CM).^10^ The researchers reported that 80% of the patients had OAEs, but that they disappeared over time in 11% of their patients. OAEs were not recorded in the other 9%.^24^ Raveh reported that in three of their patients, OAEs were absent and CM was present.^10^ In our study, we found that in 34 of our patients OAEs were absent and CM was present bilaterally, and in six of our patients OAEs were absent and CM was present unilaterally.

In cochlear hearing loss OAEs and CM are absent and, subsequently, the ABR threshold is elevated. However, in ANSD, the ABRs are abnormal or absent, whereas the OAEs and/or CM are normal. Cases of absent ABRs in the presence of mild to moderate hearing loss were reported in some series.^3^, ^10^ The ABRs in ANSD which are worse are accepted in cochlear SNHL, most probably because the synchrony is insufficient to evoke auditory brainstem neural activity.^24^

It was reported that the majority of patients with ANSD have absent acoustic reflex, with about 20% having highly atypically (elevated) acoustic reflex both ipsilaterally and contralaterally, but have recordable acoustic reflex to tactile stimulation.^24^ In our study, the patients with ANSD had no acoustic reflexes.

The hearing loss in ANSD may range from mild to profound and audiograms vary widely; no predominant pattern has been detected.^25^, ^26^ The degree of hearing loss in patients with the diagnosis of auditory neuropathy varied from slight to profound; most losses were bilateral and symmetrical in configuration (82%), with few patients having normal hearing in both ears and a unilateral disorder.^24^ In our study, most of the patients (12% severe–48% profound) had severe to profound hearing loss. Two (5%) of the patients with ANSD had normal hearing level.

Given such an incipient understanding of ANSD, amplification and auditory rehabilitation for these patients is challenging. For pediatric patients a comprehensive, multidisciplinary approach to the management of ANSD is recommended. A trial of hearing aid amplification should be performed depending on the audiological information. FM system can also be tried. FM systems are recommended in both ANSD patients with normal hearing and hearing aid and cochlear implant users because of the improved speech understanding in noisy conditions. Therefore, it is believed to increase success in education. In addition, at the time the cochlear implant team should evaluate the patient and then determine the candidacy for cochlear implantation based on the audiological findings, hearing aid benefit, status of speech language development and overall development skills.^27^ In our study, in 15 of the patients with ANSD who had profound hearing loss, cochlear implants were fitted; 23 of the patients with ANSD have been observed through their hearing aids and the remaining two who had normal hearing levels were applied the FM system, but their parents did not want them to use it.

Auditory rehabilitation with hearing aids is difficult for patients with ANSD because of the poor word recognition.^15^ Nevertheless, hearing aids with or without the FM system should be first stage of management. In some cases in which the pure tone hearing loss is too small or there is a normal hearing level for amplification such as our patient who had normal hearing level, the FM system can be performed or a low gain hearing aid can be used to increase auditory synchronization. In Raveh's series, only one in 19 patients had useful amplification and speech development.^10^ Rance and et al. demonstrated that nearly 50% of children affected with ANSD benefitted from amplification.^26^ In our study, the significant improvement was observed between before the use of hearing aids and after the use of hearing aids.

Cochlear implant provides supraphysiologic electrical stimulation to the auditory nerve and may improve the synchronicity of the neural activity.^10^ Studies have reported improved audiological performance; good implants evoked brainstem responses, as well as good NRT after implantation.^10^, ^15^, ^23^, ^28^ Fifteen of our patients who failed hearing aid management were fitted with cochlear implants. Intraoperative and post operative NRTs were within normal limits. All patients with sufficient follow-up to date demonstrated significant improvement. However, we observed that statistically there was not a difference between the use of hearing aids and the use of cochlear implantation groups. The reason was that all of the cochlear implantation users with ANSD had severe to profound hearing loss as the other CI users with SNHL. It was similar to the hearing aid users, because most of them had mild or moderate hearing loss. Thus, we could not find auditory perception or language development difference in outcomes between children with ANSD and children with SNHL who use HA or CI.

## Conclusion

Comprehensive audiological evaluation is required for accurate diagnosis. The best management of ANSD is provided by a multidisciplinary approach, and amplification preferences should be chosen depending on the hearing loss degree and language development as SNHL.

## Authorship

Çağıl Gökdoğan was in charge of data collection, analysis and the writing of the paper; Şenay Altınyay was in charge of data collection, analysis and the writing of the paper; Bülent Gündüz was in charge of data collection, analysis and the writing of the paper; Yusuf Kemal Kemaloğlu was in charge of the writing of the paper; Yıldırım Bayazıt was in charge of the writing of the paper, and Kemal Uygur was in charge of the writing of the paper.

## Conflicts of interest

The authors declare no conflicts of interest.
